# Virome Analysis Reveals Diverse and Divergent RNA Viruses in Wild Insect Pollinators in Beijing, China

**DOI:** 10.3390/v14020227

**Published:** 2022-01-24

**Authors:** Nannan Li, Yizhao Huang, Wei Li, Shufa Xu

**Affiliations:** 1 Institute of Apicultural Research, Chinese Academy of Agricultural Sciences, Beijing 100093, China; linan_caas@163.com; 2 College of Food Science and Pharmaceutical Engineering, Zaozhuang University, Zaozhuang 277160, China; 18763890989@163.com

**Keywords:** wild insect pollinators, honeybees, next-generation sequencing, novel RNA viruses, viral diversity

## Abstract

Insect pollinators provide major pollination services for wild plants and crops. Honeybee viruses can cause serious damage to honeybee colonies. However, viruses of other wild pollinating insects have yet to be fully explored. In the present study, we used RNA sequencing to investigate the viral diversity of 50 species of wild pollinating insects. A total of 3 pathogenic honeybee viruses, 8 previously reported viruses, and 26 novel viruses were identified in sequenced samples. Among these, 7 novel viruses were shown to be closely related to honeybee pathogenic viruses, and 4 were determined to have potential pathogenicity for their hosts. The viruses detected in wild insect pollinators were mainly from the order *Picornavirales* and the families *Orthomyxoviridae*, *Sinhaliviridae*, *Rhabdoviridae*, and *Flaviviridae*. Our study expanded the species range of known insect pollinator viruses, contributing to future efforts to protect economic honeybees and wild pollinating insects.

## 1. Introduction

Insect pollinators are necessary for most flowering plants, playing a key role in both wild plant reproduction and food production security [1,2]. Pollinating insects have a large species range, mainly belonging to three orders (i.e., Hymenoptera, Diptera, and Lepidoptera [3]. Pollinator biodiversity is critical for pollination quality in agricultural productivity and the conservation of the ecosystem [4,5], but the number of insect pollinators, including honeybees, is declining worldwide [6,7]. Several factors underlying pollinator decline are: heavy use of pesticides; worldwide spread of parasites, especially *Varroa destructor*; diseases caused by pathogenic viruses and other pathogens; monoculture cropping and plant biodiversity reduction; and competition between native and invasive species [6,8,9]. Additionally, many of these factors act simultaneously on insect pollinators and, due to their concurrent relationship, can exert additive or even synergistic harmful effects [6]. In the case of honeybees, several factors leading to the outbreak of viral diseases have already been identified: *V. destructor* parasitism [10], low-quality food [11], exposure to pesticides, and so forth [12,13,14,15,16,17].

Commercial pollination services have significantly contributed to the growth of agricultural production [18,19,20]. To protect the population of pollinators and avoid the spread of viruses, virus detection can help in designing a better management program to prevent the transmission of viruses from commercial insect pollinators to wild insect pollinators. The widespread application of next-generation sequencing technology in virus research has revealed a large number of novel viruses and contributed to the understanding of both the distribution and the evolutionary relationship of viruses [21,22]. For instance, the recent discovery of rhabdoviruses and flaviviruses in honeybees is based on sequencing technology [23]. Forty-two novel viruses in the order *Picornavirales* associated with *Apis mellifera* have been detected by RNA sequencing (RNA-seq) [24], and an orthomyxovirus has been detected in *A. mellifera* and *V. destructor* by using transcriptome sequencing. Detection of positive-sense RNA strands subsequently confirmed that this virus can infect both *V. destructor* and *A. mellifera* [25].

An RNA virus of an invertebrate was redefined by profiling the viromics of over 220 invertebrate species sampled across nine animal phyla; identified viruses fill major gaps in the RNA virus phylogeny and reveal an evolutionary history that is characterized by both host switching and codivergence [21]. Bees are ancient insects, and the evolution of viruses carried by them may provide us with a new perspective to understand the evolution of insect viruses [23]. Though many studies have focused on the diversity of honeybee viruses, their evolutionary relationships still need further research. For example, while the Lake Sinai virus (LSV) has been classified as *Nodamuvirales*, the LSV-containing clade has been shown to be distinct from the *Nodaviridae* family in an unrooted phylogenetic tree [26,27]. Interestingly, although Hypera postica-associated sinaivirus in the *Sinhaliviridae* family has been detected in *Hypera postica*, this virus is most closely related to the LSV [28]. Therefore, a study of viruses in host-related species has the potential to clarify as-yet-undefined viral evolutionary relationships.

Previous studies of insect pollinator viruses have primarily focused on individual viruses in honeybees [13,29,30]. Honeybee viruses transmitting to wild pollinating insects is a common phenomenon [31]. Deformed wing virus (DWV) has been proved to be able to infect wild bumblebees and cause wing deformities, and acute bee paralysis virus (ABPV), black queen cell virus (BQCV), and chronic bee paralysis virus (CBPV) were also detected in bumblebee species [32,33]. Understanding the diversity and composition of viruses in the category of pollinators as a whole can enable the development of more effective practices for the monitoring and control of the spread of viral diseases in pollinators [25,34,35]. In the present study, we employed next-generation sequencing technology for the first time to investigate viruses in wild insect pollinators and increase understanding of the diversity and evolutionary relationships of viruses in wild pollinating insects.

## 2. Materials and Methods

### 2.1. Sample Collection and Processing

Fifty specimens of wild insect pollinators (Hymenoptera: 38, Diptera: 10, and Amphiesmenoptera: 2) presenting no overt symptoms of viral diseases in the field were collected by an insect net on or next to flowering plants in Xiangshan, Beijing, from August to October 2020. The species of the specimens were identified according to their morphology, and all specimens were identified to the family level. These specimens were immediately stored in 5 mL centrifuge tubes in an ice box after capture and transferred to a refrigerator and maintained at −80 °C until processing. Specimens were then individually ground into powder in liquid nitrogen and divided into Pool A and Pool B for subsequent sequencing. Pool A contained Apidae: 10, Halictidae: 5, Syrphidae: 5, Ichneumonidae: 2, Megachilidae: 2, Asilidae: 1, Bombyliidae: 1, Calliphoridae: 1, Lycaenidae: 1, Sphingidae: 1, Tabanidae: 1, Tachinidae: 1, and Tenthredinidae: 1. Pool B contained Vespidae: 9, Sphecidae: 3, Scoliidae: 2, Apidae: 1, Halictidae: 1, Ichneumonidae: 1, and Pompilidae: 1. A complete list of specimens can be found in Supplementary Table S1.

### 2.2. Total RNA Extraction and Sequencing

The total RNA of the two mixed pools was extracted using the RNApure Total RNA Kit (RN0302; Aidlab Biotechnologies Co., Ltd., Beijing, China), according to the manufacturer’s recommendations. Sequencing libraries were generated using the VAHTS mRNA-seq v2 Library Prep Kit for Illumina (NR601-01; Vazyme, Nanjing, China), following the manufacturer’s recommendations, and index codes were added to attribute sequences to each sample. The libraries were sequenced on an Illumina NovaSeq platform to generate 150-bp paired-end reads, according to the manufacturer’s recommendations. We obtained a total of 37 Gb clean reads.

### 2.3. Sequence Assembly and Virus Detection

Sequencing reads were de novo assembled using Trinity (version 2.12.0) [34]. The contigs were compared with the GenBank nonredundant (NR) database with Diamond (version 2.0.8.146) [35]. We used a Python script to filter out nonvirus contigs, while viruslike contigs were mapped using Bowtie 2 (version 2.4.2) [36] to evaluate the contig quantity. Viral genome assembly results were manually corrected, and consensus sequences were generated by Geneious Primer (version 2020.0.4). Virus open reading frames (ORFs) were annotated based on the results of ORFfinder (version 0.4.3) [37] and the structure of the most closely related viral genome. Conserved domains of new viruses were identified using NCBI conserved domain database BLAST searches (version 2.11.0) [38].

### 2.4. Phylogenetic Analyses

Protein sequences of representatives from all viral species, as proposed by the International Committee on Taxonomy of Viruses (ICTV), were collected from GenBank and used for phylogenetic analyses. The RdRp region of novel viruses was used to retrieve related ICTV-classified viruses by BlastP. The RdRp domains of novel viruses and homologous viral proteins were aligned using MUSCLE (version 3.8.31) [39,40]. All ambiguously aligned regions were subsequently removed using Trimal (version 1.2rev59) [41]. The best-fit model of amino acid substitution in each dataset was determined using ModelTest [42]. Phylogenetic trees were inferred using the maximum likelihood method implemented in RAxML with 1000 bootstrap replicates [43]. Phylogenetic trees were displayed and annotated using FigTree (version 1.4.4). Pairwise sequence comparisons were performed by using MUSCLE to align the pairwise combinations of viral RdRp regions in each group and calculate the identity value of each alignment. The numerical matrix of each group was displayed by a heat map using the pheatmap package (version 1.0.12), and the pairwise sequence comparison results were used to verify the classification results of novel viruses based on their evolutionary trees.

### 2.5. Identification of Virus Hosts

We extracted RNA from each specimen powder sample before mixing using the RNApure Total RNA Kit (Aidlab). cDNA was prepared using the TaKaRa PrimeScript RT Reagent Kit (Perfect Real Time RR037A; Takara Biomedical Technology Co., Ltd., Beijing, China). The presence of a virus was determined using reverse transcription-polymerase chain reaction (RT-PCR), which was carried out with the first-strand cDNA products using the 2 × TSINGKE Master Mix (blue) (TSE004; Beijing TsingKe Biotech Co., Ltd., Beijing, China) in 25 µL reactions and specific primers. The specific primers were designed based on the assembled viral genome sequences (Supplementary Table S2). PCR products were confirmed for the presence of a target fragment by agarose gel electrophoresis and DNA sequencing.

### 2.6. Strand-Specific Detection of Novel Viruses

For identifying the reverse complementary strand of the novel viruses, the strand-specific primers (Supplementary Table S2) were designed in conformity with the method of a previous study, with slight modifications [44]. Specific primers were designed based on the assembled viral genome sequences, and a tag sequence (TCATGGTGGCGAATAA) was added to the forward primer. In reverse transcription, RNA was reverse-transcribed with tagged forward primers, and the control group with normal, random primers. Then the tag sequence as the forward primer and normal reverse primer was used for PCR detection of cDNA products. PCR products were confirmed for the presence of a target fragment by agarose gel electrophoresis and DNA sequencing.

## 3. Results

### 3.1. Identification of Known Viruses in Wild Pollinators

The Trinity software was used to assemble the sequencing data, and the Diamond program was used to annotate contigs before querying the GenBank NR database. In the present study, in total, eight known viruses were identified in the annotated results. The related contigs were further assembled to obtain consensus sequences (Table 1). Among these eight viruses, DWV, CBPV, and ABPV were found to be widely distributed honeybee pathogenic viruses; the Mayfield virus 1, previously detected in *Bombus terrestris* in Lebanon and the UK; the Vespa velutina-associated acypi-like virus, previously detected in *Vespa velutina nigrithorax* in France; the Scaldis River bee virus, previously detected in *Osmia cornuta* in Belgium; the Arboretum almendravirus, previously detected in the mosquito *Psorophora albigenu* in Peru; and the Hubei Diptera virus 6, previously detected in Diptera in China.

### 3.2. Identification of Novel RNA Viruses

**Figure 1:**
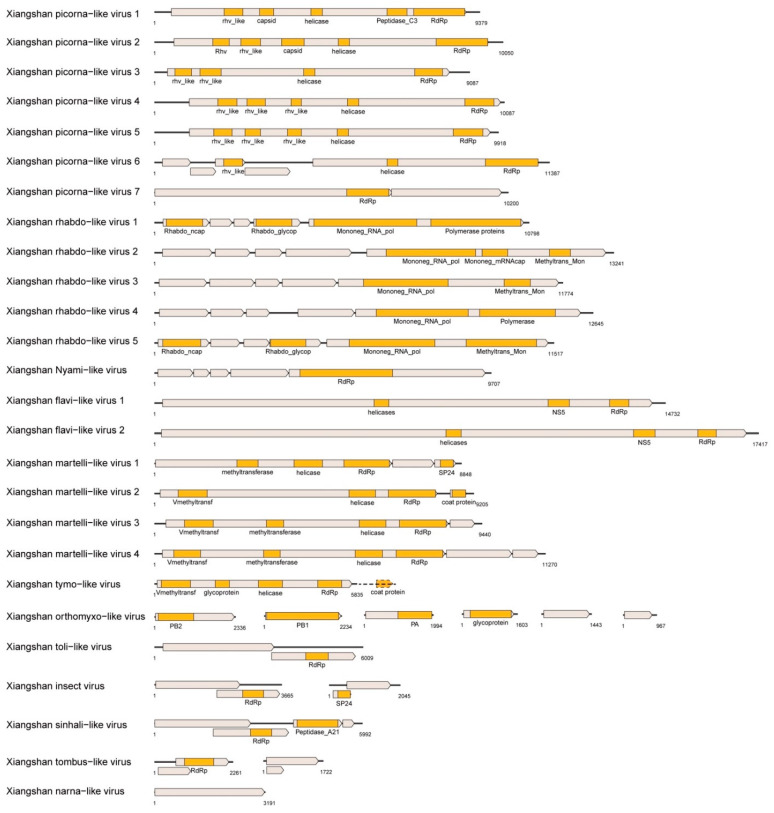
Genome structures of novel viruses. Conservative domains (represented by brown rectangles) were annotated by CDD, while ORFs (represented by gray rectangles) were annotated based on the results of ORFfinder and the structure of the most closely related viral genome. The Xiangshan tymo-like virus may have an unassembled ORF encode capsid protein, which is represented by a dotted line.

**Figure 2:**
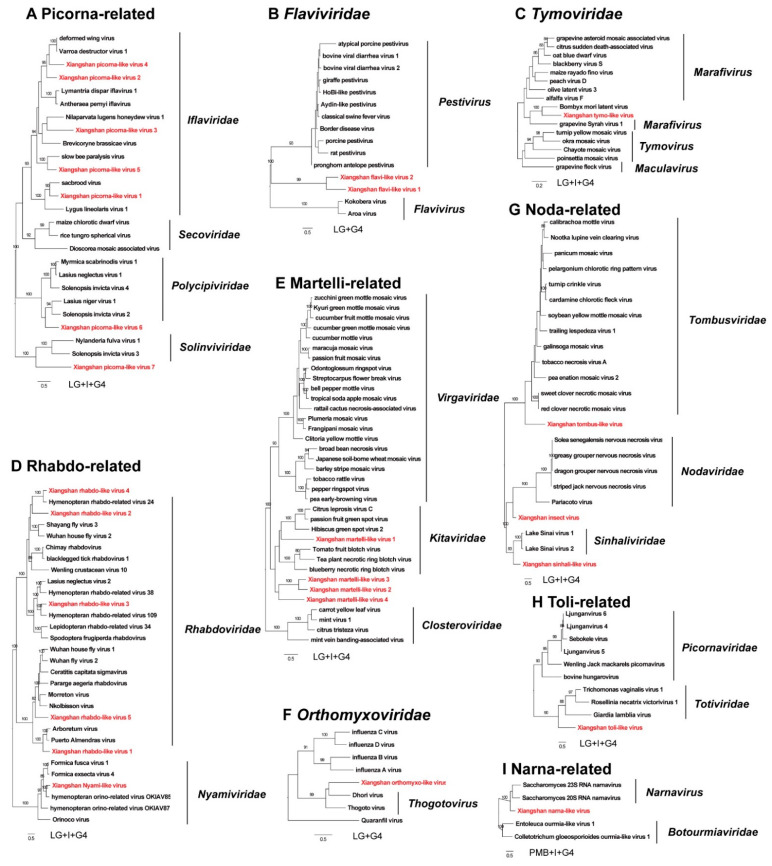
Phylogenetic relationship of novel viruses. The phylogenetic relationship of novel viruses was inferred from conserved RdRp amino acid sequences. Related viruses only include those accurately classified by ICTV. RdRp-conserved sequences of novel viruses and related viruses in the trees were identified by CDD. Midpoint rooting phylogenetic trees were built using the maximum likelihood method with a bootstrap value of 1000, and bootstrap values (>80%) were shown on the branches. The amino acid substitution model was annotated below each tree. The tree of Orthomyxoviridae was based on polymerase subunit PA amino acid sequences, and the tree of narnavirus was based on complete RdRp amino acid sequences.

A total of 26 novel RNA viruses were identified in this study (Table 2). ORFs of novel viruses were identified by the ORFfinder software and referenced to the genome structure of closely related viruses (Figure 1). Compared with the related viruses, ORFs of all novel viruses were complete or nearly complete. RdRp regions of the novel viruses were checked with the online BLASTp tool. The identity value with the most closely related viruses ranged from 34.8% to 73.9%, indicating that these viruses were significantly different from known viruses. The phylogenetic tree constructed using the RdRp region (Figure 2) and the pairwise identity comparison (Supplementary Figure S1) in each group showed that 21 novel viruses could be divided into 9 virus families: *Iflaviridae*: 5 (Figure 2A), *Polycipiviridae*: 1 (Figure 2A), *Flaviviridae*: 2 (Figure 2B), *Tymoviridae*: 1 (Figure 2C), *Rhabdoviridae*: 5 (Figure 2D), *Nyamiviridae*: 1 (Figure 2D), *Kitaviridae*: 4 (Figure 2E), *Orthomyxoviridae*: 1 (Figure 2F), *Sinhaliviridae*: 1 (Figure 2G).

Phylogenetic analysis showed that seven novel viruses have closer relationships with honeybee pathogenic viruses than other viruses within the tree, determined using multiple sequence alignment comparison by the MUSCLE program. Of the seven novel viruses, four novel viruses in the order *Picornavirales* were similar to honeybee pathogenic viruses. XPLV2 and XPLV4 share 45.94% and 46.39% amino acid identity with DWV (NC_004830.2), respectively. XPLV5 share 41.4% amino acid identity with slow bee paralysis virus (SBPV, NC_014137.1). XPLV1 share 47.26% amino acid identity with Sacbrood virus (SBV, NC_002066.1). A novel virus in Orthomyxoviridae, the Xiangshan orthomyxo-like virus (XOLV), share 34.98% amino acid identity in polymerase subunit PA with Varroa orthomyxovirus-1 (VOV-1, MK032467.1). The Xiangshan insect virus (XIV) and the Xiangshan sinhali-like virus (XSLV) share 35.1% and 38.26% amino acid identity in RdRp with CBPV (NC_010711.1) and LSV (NC_032433.1), respectively.

### 3.3. Identification of Complementary Strand of Novel Viruses

**Figure 3:**
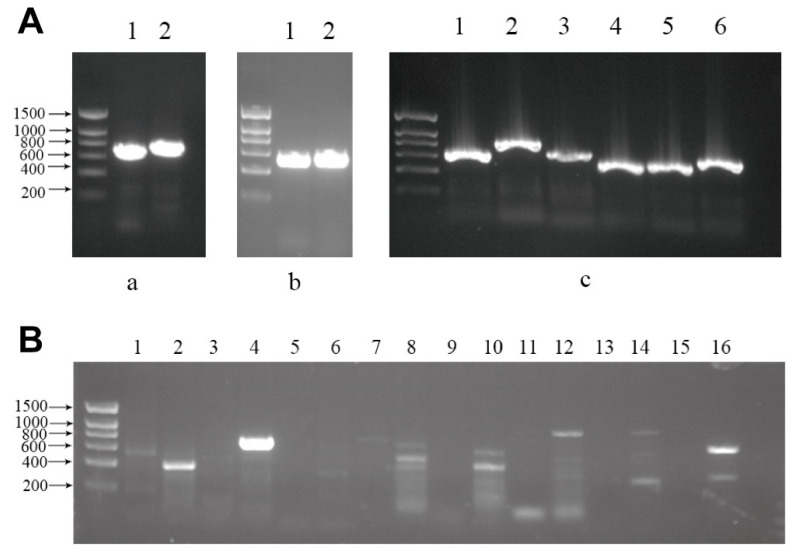
(**A**) Detection of each segment of segmented RNA viruses. (**a**) Xiangshan tombus-like virus segments 1 and 2 correspond to lanes 1 and 2. (**b**) Xiangshan insect virus segments 1 and 2 correspond to lanes 1 and 2. (**c**) Xiangshan orthomyxo-like virus segments 1–6 correspond to lanes 1–6. All PCR products were further verified by DNA sequencing. (**B**) Detection of complementary strands of several novel viruses. XOLV (lanes 3, 4), XTLV (lanes 5, 6), XPLV1 (lanes 7, 8), XPLV2 (lanes 9, 10), XSLV (lanes 11, 12), XIV (lanes 13, 14), and XPLV4 (lanes 15, 16). Lanes 3, 5, 7, 9, 11, 13, and 15 were the respective controls. DNA sequencing of PCR products of lanes 4, 8, 12, and 16 shows these bands in the specific amplification of virus sequences.

We selected seven novel viruses (XOLV, XTLV, XPLV2, XIV, XSLV, XPLV1, and XPLV4) to identify their reverse complementary chain to the host and used RT-PCR-tested insect samples to confirm the hosts of these seven viruses (Table 3). We further confirmed that these segments came from one sample by designing specific primers for each segment of three RNA viruses with segmented genomes (Figure 3A). Strand-specific RT-PCRs were employed to investigate whether the virus could replicate in the hosts. We screened for the presence of the positive-sense RNA strand of negative-sense genome viruses and the negative-sense RNA strand of positive-sense genome viruses. Specific primer information is listed in Supplementary Table S2. Results showed reverse complementary strands of XOLV, XPLV1, XSLV, and XPLV4 in the corresponding host samples, indicating that these viruses can replicate within and might be pathogenic to the hosts (Figure 3B).

## 4. Discussion

RNA-seq is a powerful tool that has been widely used to analyze the composition of known viruses, discover novel RNA viruses, and detect pathogenic viruses [22,45,46]. Many studies have focused on the viromics of honeybees using sequencing methods, significantly improving the scientific understanding of bee viruses [23,26,47,48,49,50]. Far fewer studies have been performed on wild insect pollinators, although their survival is also threatened by viruses [7,51,52,53]. By using the RNA-seq method in the present study, we performed the first preliminary exploration of the viromic characteristics of wild insect pollinators collected from flowering plants in Xiangshan, Beijing, China.

In the present study, DWV, ABPV, and CBPV were detected in pool B (which did not include *Apis* species), indicating that the three honeybee pathogenic viruses can also inhabit non-*Apis* wild insect pollinators. Previous studies have indicated that DWV and BQCV are able to replicate their genomes in bumblebees [53,54,55]. Therefore, it is confirmed that honeybee viruses can spread to other pollinators of the same family [56].

In this study, 5 previously reported insect viruses, the Mayfield virus 1 (host *B. terrestris*), the Vespa velutina-associated acypi-like virus (host *V. velutina nigrithorax*), the Scaldis River bee virus (host *O. cornuta*), the Hubei Diptera virus 6 (host Diptera), and the Arboretum almendravirus (host *P. albigenu*) were also detected in our samples, indicating that they may be prevalent and have been distributed in the corresponding hosts [21,57,58,59]. The Scaldis River bee virus and Arboretum almendravirus detected in this study shared 74.88% and 77.56% nucleotide identity with the reference sequences, but they shared higher amino acid identity in L protein (81% and 90.7%). In terms of similarity, they might not be classified as a new species. In the current study, they were classified as an “isolate” of the reference viruses.

We confirmed the existence of 26 novel RNA viruses by assembling sequencing data. Previous studies reported that bee-infecting viruses were primarily positive-sense single-stranded ssRNA (+ssRNA) viruses of the order *Picornavirales* [24,60]. Our study showed that, of the 26 identified novel viruses, 7 can be classified in the order *Picornavirales*, suggesting that *Picornavirales* viruses are ubiquitous in insect pollinators. Previous studies also suggested that viruses in the families *Rhabdoviridae*, *Flaviviridae*, *Orthomyxoviridae*, and *Sinhaliviridae* can infect honeybees [23,61]. Our study here also showed that novel viruses in wild pollinators belong to these families, suggesting that categories of viruses in wild insect pollinators are similar to categories of viruses found in honeybees. Moreover, 5 rhabdoviruses were detected in this study, clearly indicating that rhabdoviruses are more common in wild insect pollinators. Previous studies reported that the *Kitaviridae* family consists of plant viruses [62]. Four novel viruses belonging to this family were detected in this study, and we think these 4 viruses might originate from the plant’s pollen from which we collected our specimens. Seven novel viruses (XPLV2, XPLV4, XPLV5, XPLV1, XSLV, XIV, and XOLV) showed high amino acid sequence similarity with honeybee pathogenic viruses (DWV, SBPV, SBV, LSV, CBPV, and VOV-1). Single-stranded RNA (ssRNA) viruses produce reverse complementary strands during their replication in hosts. Several studies have already proved that an ssRNA virus can replicate in the host when the reverse complementary strand of the ssRNA virus is present [25,34,54,63]. By detecting the complementary strand of seven novel viral positive- or negative-sense RNA, 4 novel viruses (XOLV, XPLV1, XSLV, and XPLV4) could replicate in their corresponding host, which suggests that these viruses present the risk of virulence to their hosts and can closely interact with honeybees in a shared ecosystem [64].

## 5. Conclusions

We detected 26 novel RNA viruses in wild insect pollinators using RNA-seq, largely expanding previous understanding of the species range of insect pollinator viruses and their interspecies virulence risk and revealing the diversity of the virus composition of wild pollinating insects. These findings have applications for efforts to protect pollinator populations and ecosystems going forward, adding to knowledge of the risks faced by not only honeybees but all insect pollinators. Furthermore, this work suggests that there are still many undiscovered viruses in wild insect pollinators, indicating that more research is called for in this field.

## Figures and Tables

**Table 1:** Identified known viruses in wild pollinators.

Virus Name	Pool	Contig Length	Read Number	Read Coverage	Closest Nucleotide Accession	Query Coverage (%)	Nucleotide Identity (%)
DWV	A	8948	33,345	100	AB070959.1	100	96.65
	B	10,086	58,750	100	MH267695.1	100	96.49
Mayfield virus 1	A	9024	13,680	100	MH614304.1	96	94.94
	B	7668	554	100	MH614304.1	96	94.4
Vespa velutina-associated acypi-like virus	A	9919	249,864	100	MN565043.1	98	94.64
	B	9877	91,996	100	MN565043.1	98	95
Scaldis River bee virus	A	7842	694	100	KY053857.1	94	74.88
Hubei Diptera virus 6 RNA1	A	6406	8923	100	KX884805.1	99	92.54
Hubei Diptera virus 6 RNA2	A	2089	2510	96.7	KX884806.1	96	92.32
Arboretum almendravirus	A	11,492	30,383	100	KC994644.1	100	77.56
ABPV	B	9481	2598	100	MN565031.1	99	96.78
CBPV RNA1	B	3592	332	100	KX168412.1	100	97.88
CBPV RNA2	B	2349	228	100	MF175174.1	95	98.44

Read number and mapping coverage were obtained using Bowtie 2, and mapped sequence reads were used to assemble contigs. The online BLASTn tool was used to compare the assembled viral contig sequences with sequences in the database. Closest nucleotide accessions were came from GenBank.

**Table 2:** Identified novel viruses in wild pollinators.

Virus Name	Pool	Family	Genus	Contig Size (bp)	Query Coverage	Subject Accession	Closest Relative (RdRp Amino Acid Identity)
Xiangshan martelli-like virus 1	A	*Kitaviridae*	Unclassified	8848	98.82	QOJ43136	Sandewavirus dungfly (67.1)
Xiangshan martelli-like virus 2	A	*Kitaviridae*	Unclassified	9205	100	QTW97796	Riboviria sp. (51.8)
Xiangshan martelli-like virus 3	B	*Kitaviridae*	Unclassified	9440	54.5	YP_009337423	Hubei virga-like virus 1 (54.5)
Xiangshan martelli-like virus 4	B	*Kitaviridae*	Unclassified	11,269	99.81	YP_009337693	Hubei virga-like virus 15 (52.1)
Xiangshan orthomyxo-like virus	A	*Orthomyxoviridae*	Unclassified	2336 + 2233 + 1994 + 1604 + 1443 + 967	99.84	QOQ34681	Dhori thogotovirus (subunit PA: 36.2)
Xiangshan tombus-like virus	A	Unclassified	Unclassified	2261 + 1722	100	QED21532	Cushing virus (64.7)
Xiangshan insect virus	B	Unclassified	Unclassified	3665 + 2045	100	YP_009011225	Anopheline-associated C virus (49.7)
Xiangshan sinhali-like virus	B	*Sinhaliviridae*	Unclassified	5770	100	ASS83305	Lake Sinai virus (49.7)
Xiangshan picorna-like virus 1	A	*Iflaviridae*	*Iflavirus*	9379	99.46	QQX28927	Soybean thrips ifla-like virus 10 (60.7)
Xiangshan picorna-like virus 2	A	*Iflaviridae*	*Iflavirus*	10,050	98.92	QKW94218	PNG bee virus 13 (67.8)
Xiangshan picorna-like virus 3	B	*Iflaviridae*	*Iflavirus*	9087	97.28	QPI13031	Iflavirus IricIV-4 (50.1)
Xiangshan picorna-like virus 4	B	*Iflaviridae*	*Iflavirus*	10,087	99.47	AWK77848	Darwin bee virus 3 (56.3)
Xiangshan picorna-like virus 5	B	*Iflaviridae*	*Iflavirus*	9918	100	YP_009337760	Hubei odonate virus 4 (69.0)
Xiangshan picorna-like virus 6	A	*Polycipiviridae*	*Sopolycivirus*	11,387	99.28	AXA52568	Linepithema humile polycipivirus 1 (51.2)
Xiangshan picorna-like virus 7	A	Unclassified	Unclassified	10,200	85.95	QIT20099	Diabrotica undecimpunctata virus 1 (38.5)
Xiangshan rhabdo-like virus 1	A	*Rhabdoviridae*	Unclassified	10,798	98.74	YP_009094394	Puerto Almendras virus (47.1)
Xiangshan rhabdo-like virus 2	A	*Rhabdoviridae*	Unclassified	13,241	92.08	AJG39108	Jingshan fly virus 2 (34.8)
Xiangshan rhabdo-like virus 3	A	*Rhabdoviridae*	*Alphahymrhavirus*	11,774	99.06	QMP82144	Hymenopteran rhabdo-related virus OKIAV109 (51.5)
Xiangshan rhabdo-like virus 4	B	*Rhabdoviridae*	*Betahymrhavirus*	12,645	99.77	QPB73983	Hymenopteran rhabdo-related virus OKIAV24 (58.9)
Xiangshan rhabdo-like virus 5	B	*Rhabdoviridae*	Unclassified	11,517	99.11	QMP82217	Lepidopteran rhabdo-related virus OKIAV3 (47.8)
Xiangshan nyami-like virus	B	*Nyamiviridae*	*Formivirus*	9707	99.15	QPB73978	Hymenopteran orino-related virus OKIAV85 (63.5)
Xiangshan narna-like virus	A	Unclassified	Unclassified	3191	83.7	APG77263	Wenling narna-like virus 8 (35.3)
Xiangshan tymo-like virus	B	*Tymoviridae*	*Marafivirus*	9087	100	QQG34658	Nasturtium officinale macula-like virus 1 (73.9)
Xiangshan toli-like virus	B	Unclassified	Unclassified	6009	90.87	YP_009143313	Camponotus yamaokai virus (57.3)
Xiangshan flavi-like virus 1	B	*Flaviviridae*	Unclassified	14,732	100	QTJ63564	Hymenopteran flavi-related virus (54.9)
Xiangshan flavi-like virus 2	B	*Flaviviridae*	Unclassified	17,417	100	QTJ63570	Hymenopteran flavi-related virus (58.1)

RdRp regions of novel viruses were identified using CDD, and the BLASTp online tool was used to search for the closest relative virus. The closest relative of Xiangshan orthomyxo-like virus and Xiangshan narna-like virus was searched using polymerase subunit PA aa and RdRp aa, respectively. Only the results supported both by evolutionary tree and pairwise sequence comparison were shown in classification information. Subject accessions came from GenBank. “+” in the column “Contig size” means the separator of each segment size of segmented RNA viruses. Numbers in the brackets in the column “Closest relative” mean the identity between the RdRp region of novel virus and subject sequence.

**Table 3:** Complementary strand detection of seven novel viruses.

Virus Name	Genome Type	Host	Complementary Strand Detection
Xiangshan orthomyxo-like virus	ssRNA (−)	*Eristalisi tenax* (L.)	+
Xiangshan tombus-like virus	ssRNA (+)	*Sphaerophoria indiana* Bigot	−
Xiangshan picorna-like virus 2	ssRNA (+)	*Amegilla zonata* L.	−
Xiangshan insect virus	ssRNA (+)	Vespidae	−
Xiangshan sinhali-like virus	ssRNA (+)	Sphecidae	+
Xiangshan picorna-like virus 1	ssRNA (+)	Tabanidae	+
Xiangshan picorna-like virus 4	ssRNA (+)	*Scolia sinensis Saussure* et Siehel	+

Positive results of the reverse complementary strand are denoted by ‘+’, and negative results are denoted by ‘−’.

## Data Availability

The raw sequence data used for analysis are available in NCBI under the Sequence Read Archive (SRA), BioProject No. PRJNA728541 and SRA accession numbers SRR14554108–SRR14554111. SRR14554109 and SRR14554108 are from the same library of pool B. SRR14554109 and SRR14554110 are from the same library of pool A. The sequences of detected viruses were submitted to GenBank and given accession numbers OK491477–OK491521.

## Supplementary Materials

The following are available online at https://www.mdpi.com/article/10.3390/v14020227/s1, Supplementary Figure S1: Pairwise sequence comparisons of novel viruses; Supplementary Table S1: Sample information of collected insects; Supplementary Table S2: Primer information of RT-PCR.
