# Supplementary material for: Virome Analysis Reveals Diverse and Divergent RNA Viruses in Wild Insect Pollinators in Beijing, China
Source: Viruses. 2022 Jan 24;14(2):227. doi: 10.3390/v14020227 (PMC8877953; doi:10.3390/v14020227)
Supplement: Supplementary file 1 [file viruses-14-00227-s001.zip › Supplementary Figure 1.pdf]

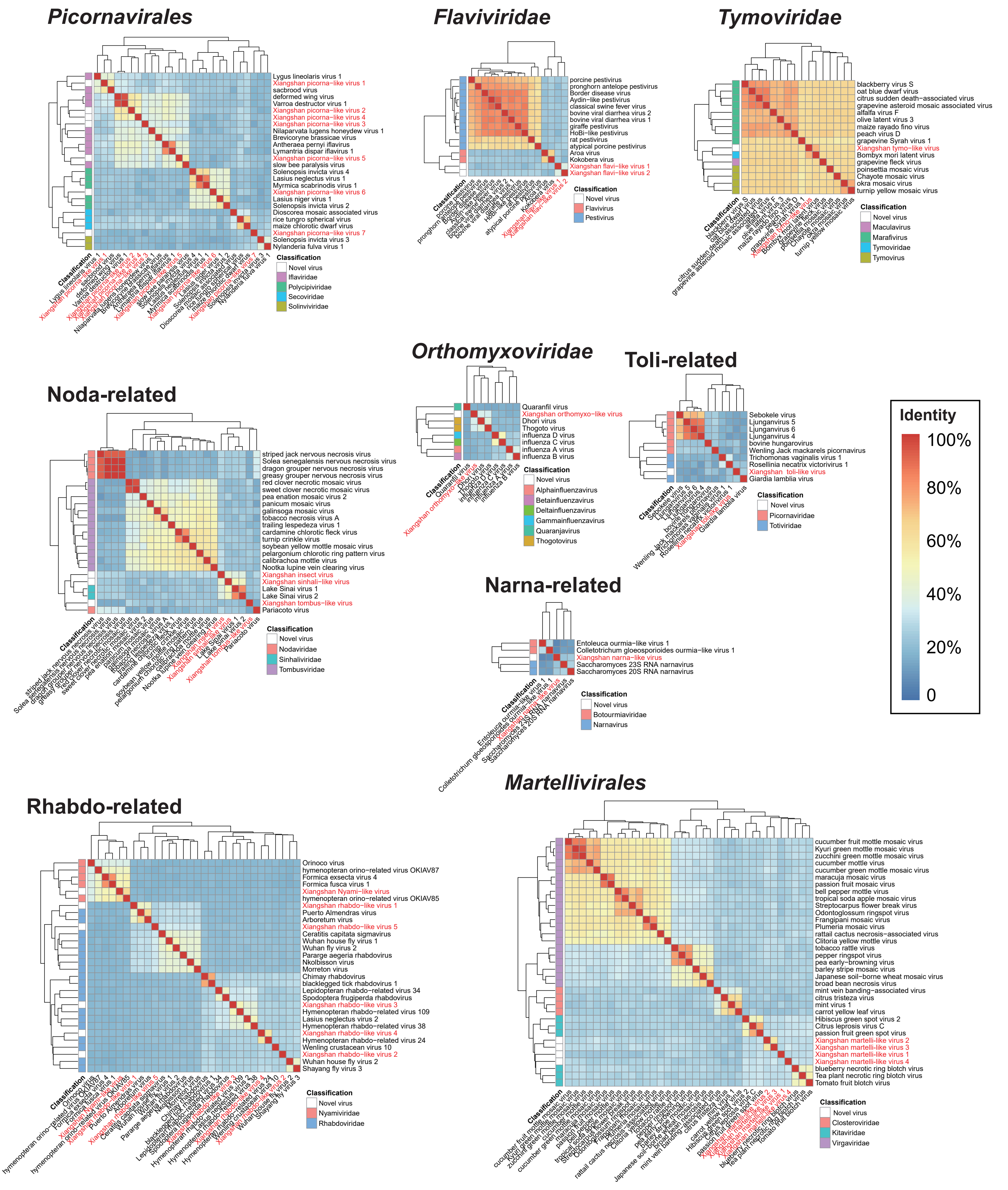
Picornavirales
Lygus lineolaris virus 1
Xiangshan picorna–like virus 1
sacbrood virus
deformed wing virus
Varroa destructor virus 1
Xiangshan picorna–like virus 2
Xiangshan picorna–like virus 4
Xiangshan picorna–like virus 3
Nilaparvata lugens honeydew virus 1
Brevicoryne brassicae virus
Antheraea pernyi iflavirus
Lymantria dispar iflavirus 1
Xiangshan picorna–like virus 5
slow bee paralysis virus
Solenopsis invicta virus 4
Lasius neglectus virus 1
Myrmica scabrinodis virus 1
Xiangshan picorna–like virus 6
Lasius niger virus 1
Solenopsis invicta virus 2
Dioscorea mosaic associated virus
rice tungro spherical virus
maize chlorotic dwarf virus
Xiangshan picorna–like virus 7
Solenopsis invicta virus 3
Nylanderia fulva virus 1
Classification
Novel virus
Iflaviridae
Polycipiviridae
Secoviridae
Solinviviridae
Flaviviridae
porcine pestivirus
pronghorn antelope pestivirus
Border disease virus
Aydin–like pestivirus
classical swine fever virus
bovine viral diarrhea virus 2
bovine viral diarrhea virus 1
giraffe pestivirus
HoBi–like pestivirus
rat pestivirus
atypical porcine pestivirus
Aroa virus
Kokobera virus
Xiangshan flavi–like virus 1
Xiangshan flavi–like virus 2
Classification
Novel virus
Flavivirus
Pestivirus
Tymoviridae
blackberry virus S
oat blue dwarf virus
citrus sudden death–associated virus
grapevine asteroid mosaic associated virus
alfalfa virus F
olive latent virus 3
maize rayado fino virus
peach virus D
grapevine Syrah virus 1
Xiangshan tymo–like virus
Bombyx mori latent virus
grapevine fleck virus
poinsettia mosaic virus
Chayote mosaic virus
okra mosaic virus
turnip yellow mosaic virus
Classification
Novel virus
Maculavirus
Marafivirus
Tymoviridae
Tymovirus
Orthomyxoviridae
Quaranfil virus
Xiangshan orthomyxo–like virus
Dhori virus
Thogoto virus
influenza D virus
influenza C virus
influenza A virus
influenza B virus
Classification
Novel virus
Alphainfluenzavirus
Betainfluenzavirus
Deltainfluenzavirus
Gammainfluenzavirus
Quaranjavirus
Thogotovirus
Toli-related
Sebokele virus
Ljunganvirus 5
Ljunganvirus 6
Ljunganvirus 4
bovine hungarovirus
Wenling Jack mackarels picornavirus
Trichomonas vaginalis virus 1
Rosellinia necatrix victorivirus 1
Xiangshan toli–like virus
Giardia lamblia virus
Classification
Novel virus
Picornaviridae
Totiviridae
Identity
100%
80%
60%
40%
20%
0
Noda-related
striped jack nervous necrosis virus
Solea senegalensis nervous necrosis virus
dragon grouper nervous necrosis virus
greasy grouper nervous necrosis virus
red clover necrotic mosaic virus
sweet clover necrotic mosaic virus
pea enation mosaic virus 2
panicum mosaic virus
galinsoga mosaic virus
tobacco necrosis virus A
trailing lespedeza virus 1
cardamine chlorotic fleck virus
turnip crinkle virus
soybean yellow mottle mosaic virus
pelargonium chlorotic ring pattern virus
calibrachoa mottle virus
Nootka lupine vein clearing virus
Xiangshan insect virus
Xiangshan sinhali–like virus
Lake Sinai virus 1
Lake Sinai virus 2
Xiangshan tombus–like virus
Pariacoto virus
Classification
Novel virus
Nodaviridae
Sinhaliviridae
Tombusviridae
Narna-related
Entoleuca ourmia–like virus 1
Colletotrichum gloeosporioides ourmia–like virus 1
Xiangshan narna–like virus
Saccharomyces 23S RNA narnavirus
Saccharomyces 20S RNA narnavirus
Classification
Novel virus
Botourmiaviridae
Narnavirus
Rhabdo-related
Orinoco virus
hymenopteran orino–related virus OKIAV87
Formica exsecta virus 4
Formica fusca virus 1
Xiangshan Nyami–like virus
hymenopteran orino–related virus OKIAV85
Xiangshan rhabdo–like virus 1
Puerto Almendras virus
Arboretum virus
Xiangshan rhabdo–like virus 5
Ceratitis capitata sigmavirus
Wuhan house fly virus 1
Wuhan fly virus 2
Pararge aegeria rhabdovirus
Nkolbisson virus
Morreton virus
Chimay rhabdovirus
blacklegged tick rhabdovirus 1
Lepidopteran rhabdo–related virus 34
Spodoptera frugiperda rhabdovirus
Xiangshan rhabdo–like virus 3
Hymenopteran rhabdo–related virus 109
Lasius neglectus virus 2
Hymenopteran rhabdo–related virus 38
Xiangshan rhabdo–like virus 4
Hymenopteran rhabdo–related virus 24
Wenling crustacean virus 10
Xiangshan rhabdo–like virus 2
Wuhan house fly virus 2
Shayang fly virus 3
Classification
Novel virus
Nyamiviridae
Rhabdoviridae
Martellivirales
cucumber fruit mottle mosaic virus
Kyuri green mottle mosaic virus
zucchini green mottle mosaic virus
cucumber mottle virus
cucumber green mottle mosaic virus
maracuja mosaic virus
passion fruit mosaic virus
bell pepper mottle virus
tropical soda apple mosaic virus
Streptocarpus flower break virus
Odontoglossum ringspot virus
Frangipani mosaic virus
Plumeria mosaic virus
rattail cactus necrosis–associated virus
Clitoria yellow mottle virus
tobacco rattle virus
pepper ringspot virus
pea early–browning virus
barley stripe mosaic virus
Japanese soil–borne wheat mosaic virus
broad bean necrosis virus
mint vein banding–associated virus
citrus tristeza virus
mint virus 1
carrot yellow leaf virus
Hibiscus green spot virus 2
Citrus leprosis virus C
passion fruit green spot virus
Xiangshan martelli–like virus 2
Xiangshan martelli–like virus 3
Xiangshan martelli–like virus 1
Xiangshan martelli–like virus 4
blueberry necrotic ring blotch virus
Tea plant necrotic ring blotch virus
Tomato fruit blotch virus
Classification
Novel virus
Closteroviridae
Kitaviridae
Virgaviridae
